# Whole genome uniparental isodisomy detected using single nucleotide polymorphism (SNP) microarray in molar pregnancy: a case report

**DOI:** 10.1186/s13039-025-00707-6

**Published:** 2025-02-25

**Authors:** Onyinye O. Okonkwo, Veronica Ortega, Sheila Kane, Galina Aldrete, Paulina Ramirez, Philip T. Valente, Gopalrao V.N. Velagaleti

**Affiliations:** 1 Departments of Pathology and Laboratory Medicine, UT-Health San Antonio, 7703 Floyd Curl Drive, San Antonio, TX 78229 USA; 2 Department of Obstetrics and Gynecology, UT-Health San Antonio, San Antonio, TX USA

**Keywords:** Complete hydatidiform mole (CHM), Uniparental disomy (UPD), Single nucleotide polymorphism (SNP) microarray, Homozygous inversion

## Abstract

**Background:**

Gestational trophoblastic neoplasms consist of complete and partial hydatidiform moles, both of which are considered aberrant conceptuses. Both conditions, complete hydatidiform mole (CHM) and partial hydatidiform mole (PHM), differ in histological characteristics, genetic origin and content and clinical features. CHM have a diploid karyotype, mostly 46,XX but lack maternal genetic contribution with all chromosomes of paternal origin. High-resolution SNP microarray testing is an efficient method used to determine the parental contribution of the genomic material in molar pregnancies and confirm the diagnosis.

**Case presentation:**

We present a case of CHM in a 43-year-old, G3P2Ab1 who presented to the emergency department with 2 episodes of heavy bleeding. Chromosome analysis showed a normal 46,XX karyotype but with a homozygous pericentric inversion on chromosome 9. High-resolution SNP microarray studies detected whole genome uniparental isodisomy.

**Conclusion:**

We present a case of CHM with homozygous pericentric inversion on chromosome 9 and whole genome uniparental isodisomy. This case illustrates the efficacy of high-resolution SNP microarray in confirming the diagnosis of CHM.

**Supplementary Information:**

The online version contains supplementary material available at 10.1186/s13039-025-00707-6.

## Background

Gestational trophoblastic neoplasms consists of complete and partial hydatidiform moles both of which are considered aberrant conceptuses. Both conditions, complete hydatidiform mole (CHM) and partial hydatidiform mole (PHM) differ in histological characteristics, genetic origin and content, and clinical features [1]. Clinically, it is important to distinguish CHM from PHM due to the potential for clinical persistence, malignant transformation, recurrence and the presence of a fetus [1]. Histological examination is often not very helpful in differentiating between CHM and PHM because the degree of trophoblastic proliferation and the proportion of hydropic villi vary in both conditions [1]. Further complicating the diagnosis, approximately 15–40% of non-molar spontaneous abortions show hydropic degeneration [1].

Since both CHM and PHM differ in their genomic content, with CHM being mostly diploid and PHM mostly triploid, conventional chromosome analysis was considered the gold standard for investigating these gestational trophoblastic neoplasms. However, the limitation of conventional chromosome analysis is that it cannot distinguish 46,XX or 46,XY CHM from 46,XX or 46,XY non-molar conceptus. Given the importance of accurate diagnosis of CHM to avoid unnecessary surveillance and postponing future pregnancies, molecular testing with short tandem repeat (STR) genotyping has become valuable adjunct to conventional chromosome analysis [2].

With the advent of high-resolution SNP microarrays, diagnosis and distinction between CHM and PHM have become easier. Approximately 3% of molar pregnancies are detected by SNP microarray. SNP microarray has the advantage of detecting molar pregnancies that are not identified with the use of standard techniques such as ultrasound, histopathological evaluation or conventional chromosome analysis [3, 4].

We report a case of molar pregnancy where conventional chromosome analysis showed a homozygous pericentric inversion on chromosome 9, which suggested possible whole genome uniparental isodisomy. High-resolution SNP microarray confirmed the presence of whole genome uniparental isodisomy and thus, established the diagnosis of CHM.

## Case report

**Fig. 1:**
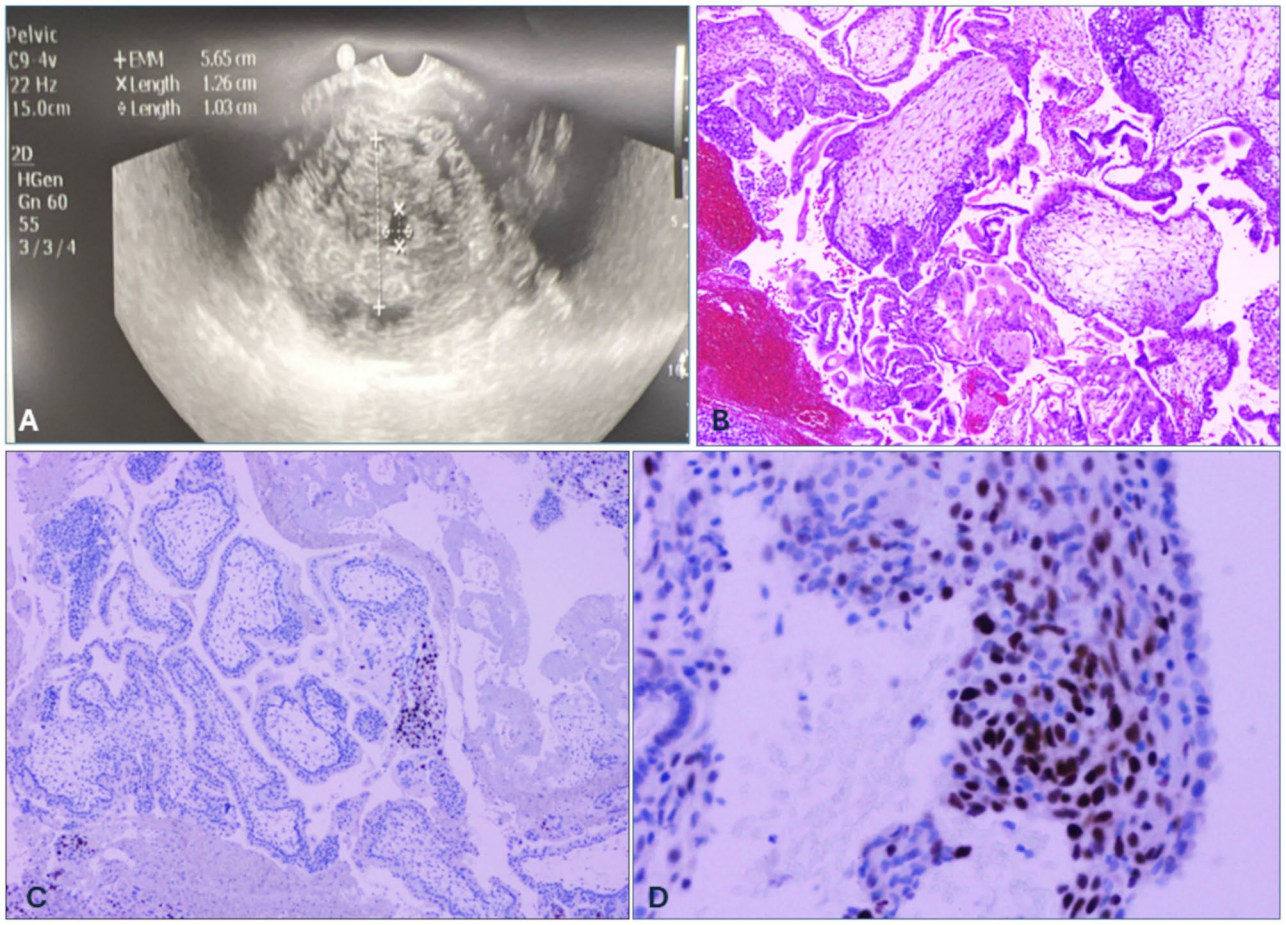
Diagnosis of molar pregnancies confirmed by (**A**). Ultrasonogram showing fluid filled spaces. (**B**). Complete hydatidiform mole: avascular hydropic villi with marked trophoblastic proliferation (H&E). (**C**). Immunostaining for p57: chorionic villi and villus trophoblasts are negative; there is focal staining of extravillus trophoblasts. (**D**). Immunostaining for p57 is positive in endometrial tissue but negative in molar trophoblasts which lack nuclear DNA of maternal origin. The positivity for p57 indicates the presence of maternal tissue; tissue of paternal origin is negative for p57. A complete mole lacks nuclear DNA of maternal origin and is negative for p57. A partial mole does contain maternal nuclear DNA and is therefore positive for p57

A 43-year-old, G3P2Ab1 presented to the emergency department with two episodes of heavy bleeding, nausea, breast soreness and a history of infertility. Pelvic examination showed a uterus measuring 14–15 weeks in size, mobile, and non-tender with no masses. Ultrasound examination showed the uterus to have fluid filled spaces (Fig. 1A). The CBC was abnormal with high neutrophil count (7.59), low red blood cell count (3.21) and low hemoglobin (10.3). The B-HCG was highly abnormal (454,572 mIU/mL). She underwent D&C for evacuation of a complete molar gestation. The patient was noted to have a plateau of the post procedure BHCG which transitioned into gradual elevation indicating an incomplete termination of the molar gestation. The patient was given chemotherapy with Actinomycin-D therapy and Depo Provera injection with 3-month dose. Baseline X-rays noted to be within normal limits with no evidence of metastatic disease at 3 months following the D&C. Further follow-up was not possible as the patient was lost for follow-up in spite of repeated efforts to contact the patient.

On histological examination, the tissue showed no discrete fetal fragments or vesicles, but hydropic chorionic villi were identified. The tissue showed focally suspended tissue fragments and avascualr hydropic villi with marked trophoblastic proliferation (Fig. 1B, Hematoxylin and Eosin stain). Immunostaining for the p57 is positive in endometrial tissue but negative in molar trophoblasts which lack nuclear DNA of maternal origin. Since CHM lack maternal nuclear DNA, immunostaining for p57 is negative in the molar trophoblasts. (Figure 1C and D). Fresh tissue samples were dissected and sent for chromosome and microarray studies. Based on these findings, a final diagnosis of CHM was determined.

**Fig. 2:**
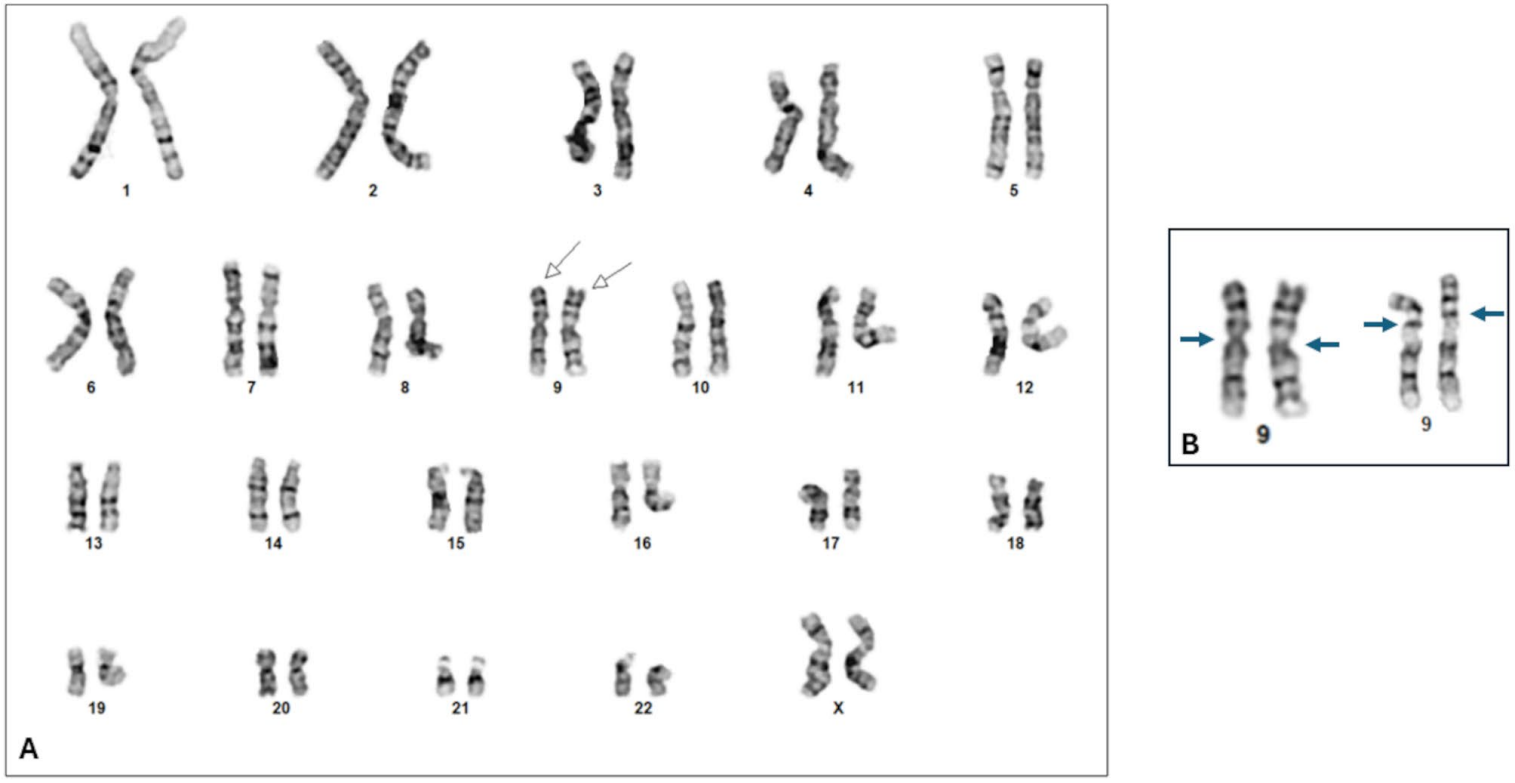
(**A**) Karyogram showing homozygous inversion from the products of conception sample. Arrows point to the chromosomes 9 with pericentric inversion. (**B**). Partial karyogram showing the homozygous inversion on chromosome 9 (left). The chromosomes 9 pair on the right is normal chromosomes 9 for comparison. The arrows show the location of the centromere clearly indicating the inversion on the pair at left

Conventional chromosome analysis was performed on the products of conception sample that included hydropic villi, residual decidua and myometrium. Culture initiation, maintenance and harvest were performed using standard methods. Chromosomes were G-banded and then analyzed using a CytoVision image analysis system (Applied Imaging, Santa Clara, CA). Analysis of 20 G-banded metaphases showed a normal 46,XX karyotype in all cells. Interestingly, all of the cells showed the presence of a homozygous pericentric inversion on chromosome 9 (Fig. 2A and B) indicating possible uniparental disomy.

High resolution SNP microarray analysis was performed as previously described [5]. In brief, chromosome microarray studies were carried out using Affymetrix CytoScan HD. The Affymetrix CytoScan^®^ HD Assay utilizes a high-density combined CGH and SNP array platform, which assesses approximately 2,696,550 markers, including approximately 750,000 SNP markers. Each oligonucleotide is approximately 25 base pairs long. Intragenic probe spacing is approximately one probe every 880 base pairs and intergenic probe spacing is approximately one probe every 1700 base pairs. To perform the assay, gDNA is digested with the Nsp1 restriction enzyme and digested DNA is then ligated to Nsp1 adapters. The ligation product is then amplified via polymerase chain reaction (PCR) to produce amplicons in the 200–1100 bp range. The amplicons are then purified and digested with DNAse I to produce 25–125 bp fragments. The fragments are end-labeled with a modified biotinylated base and the sample is hybridized to the array. The array is washed and stained with a streptavidin-coupled dye and a biotinylated anti-streptavidin antibody. The array is scanned with the Gene-Chip Scanner and the signal intensity for each marker is assessed. Using the Chromosome Analysis Suite (ChAS 3.0) software, the signal for the sample is then compared to a reference set, which is based on the average of over 400 samples. Differences in signal between the sample and reference are expressed as a log2 ratio and represents relative intensity for each marker. A discrete copy number value is determined from the relative intensity data and displayed. Genotype information for the SNP markers is visualized with the Allele Track [6].

**Fig. 3:**
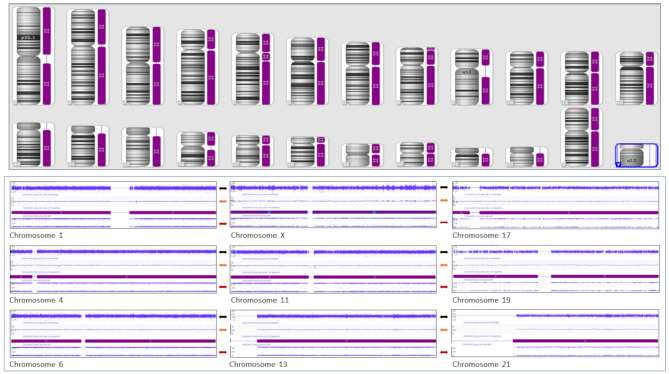
High-resolution microarray showing whole genome uniparental isodisomy. Top panel shows absence of heterozygosity (isodisomy) on every chromosome. Bottom panel showed representative chromosomes showing normal copy number profile (black double headed arrow), smooth signal at copy number of 2 (orange double headed arrow) and B-allele frequency showing complete absence of heterozygosity (isodisomy)(red double headed arrow). Only a few chromosomes (chromosomes X, 1, 4, 6, 11, 13, 17, 19 and 21) are shown in the figure due to space constrains. Please refer to supplementary Table 1 for detailed LOH data on all chromosomes

Microarray analysis showed complete homozygosity (hmz) for the entire genome, [46,XX.arr(X,1–22)x2 hmz] (Fig. 3) (Supplementary data Tables 1 and 2; supplementary Fig. 1A and 1B). This result showing complete homozygosity and the resulting uniparental isodisomy is consistent with the genetic basis of a CHM.

## Discussion

Kajii and Ohama [7] first established the genetic basis of CHM, that CHM has only a diandric paternal genome and no maternal contribution. It is estimated that 90% of CHM are monospermic where an empty egg is fertilized by a haploid 23,X sperm, and following endoreduplication, the zygote becomes diploid 46,XX. This process results in homozygous complete moles and uniparental paternal isodisomy. The remaining 10% of CHM are dispermic where an anuclear egg is fertilized by two haploid sperm resulting in diploid 46,XX or 46,XY zygote. This process results in heterozygous moles and uniparental heterodisomy [1, 8].

**Fig. 4:**
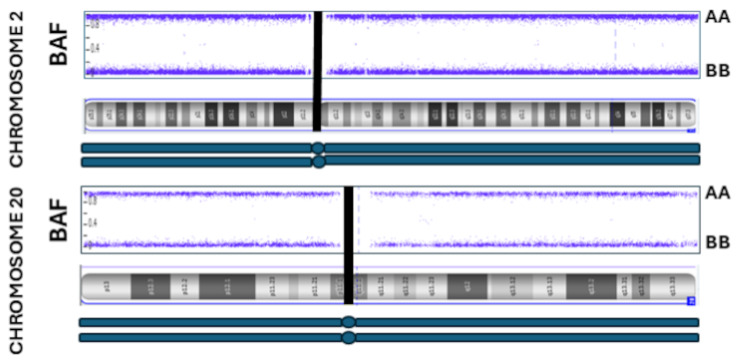
B allele frequency plot of molar pregnancy sample. Absence of heterozygosity near the centromere and throughout the chromosome is an indication of mono-haploid contribution by a single sperm origin (due to space constraints only representative chromosomes are shown)

Although the SNP microarray testing cannot determine the parental origin of the genome, the result of whole genome UPD showing isodisomy is a strong indication of monospermy and the subsequent endoreduplication of the paternal genome resulted in the molar pregnancy in our case. Given that the genetic basis of molar pregnancy is diandric diplody with no maternal genome contribution as demonstrated by the microarray result in our case, the only explanation is a diandric diploidy mechanism. In addition, the microarray result in our case falls in the first category of monospermy with a haploid 23,X sperm fertilizing an anuclear ovum and subsequent endoreduplication of the paternal genome leading to isodisomy. If the molar pregnancy in our patient is the result of dispermy, we would expect heterodisomy of some genomic content, but whole genome isodisomy further supports our hypothesis that our case is due to monospermy. As suggested by Usui et al., [9], we also looked at the B-allele frequency (BAF) plotting near the centromere to determine the zygosity status of all chromosomes. For all autosomes, the centromere zygosity status clearly demonstrated whole genome isodisomy which could only be attributed to a haploid sperm fertilizing a nullisomic egg and subsequent endoreduplication of haploid set of chromosomes resulting in diploidy [9, 10](Fig. 4). Since the recombination is suppressed near the centromere, a dispermic or heterozygous mole would be expected to show heterozygosity for loci near the centromere. While absence of such heterozygosity can also point to a meiotic 2 error, whole genome UPD due to meiotic 2 error is unlikely and the only possible explanation would be a haploid sperm fertilizing a nullisomic egg and the subsequent endoreduplication resulting in diploidy with whole genome UPD.

Although the SNP data patterns would be identical in cases of diploid eggs with duplicated haploid genomes (completely maternal origin with no paternal contribution), such a mechanism results usually in a completely different phenotypic and histologic presentation, i.e., mature cystic teratomas or fetiform teratoma [11, 12]. To our knowledge, a complete molar pregnancy with whole genome UPD of maternal origin has not been reported. As such even in the absence of STR confirmation, immunostaining by p57 supports our hypothesis that the whole genome UPD in our case is of paternal origin.

Another interesting observation in our case is the presence of homozygous pericentric inversion on chromosome 9. Pericentric inversion on chromosome 9 is considered a normal variant and the incidence is estimated to be about one– 1.65% in the general population [13]. Although homozygosity for pericentric inversion on chromosome 9 is extremely rare, few cases have been reported in the literature with the conflicting significance of such homozygosity [14]. It is estimated that the homozygous pericentric inversion 9 is expected to occur with a frequency of 1:40,000 in the general population [14]. In most cases of homozygosity for the pericentric inversion 9, the parents of the probands are consanguineous. In our case, the presence of this homozygous inversion 9 in the context of a molar pregnancy is suggestive of possible uniparental isodisomy, which was later confirmed by the SNP microarray analysis.

There are 2 previous reports of complete molar pregnancies where homozygous pericentric inversion on chromosome 9 was observed [15, 16]. Similar to our case, Kirshon et al. [15] found a homozygous pericentric inversion on chromosome 9 of paternal origin in a twin molar pregnancy and authors suggested that the presence of homozygous inversion 9 of paternal origin indicates paternal disomy. In our case, due to unavailability of the father, we could not perform chromosome analysis to conclusively prove that the homozygous inversion seen in our case is paternal in origin. On the otherhand, Abdalla and El-Kharadly [16] postulated that the inversion 9 found in both parents could be causally related to the hydatidiform moles, since the mother in their study has 3 consecutive hydatidiform moles and 4 spontaneous first-trimester miscarriages, all of them from a consanguineous marriage. However, the limitation of this study is that the authors did not actually perform any chromosome or genetic studies to determine if any of the molar pregnancies or spontaneous miscarriages have inversion 9. Since both parents are carriers of the inversion 9, there is a 25% chance that any given molar pregnancy or spontaneous abortion was homozygous for the inversion 9. As such, it is difficult to attribute that the pericentric inversion on chromosome 9 is causally related to either the molar pregnancies or spontaneous miscarriages that this consanguineous couple experienced.

There are several reports in the recent past linking the pericentric inversion on chromosome 9 to various clinical conditions including subfertility, recurrent spontaneous abortions and abnormal clinical phenotypes. However, the evidence remains controversial since most of these studies are more coincidental observations without establishing a pathogenic relationship between the inversion 9 and the observed phenotype. A recent systematic review [17] of all clinical reports with inversion on chromosome 9 concluded that there is no conclusive evidence for the pathogenecity of inversion on chromosome 9. Similarly, the recent edition of the International System of Cytogenomic Nomenclature [18] continues to categorize the pericentric inversion on chromosome 9 as common population variant.

We suggest that the homozygous pericentric inversion on chromosome 9 seen in our case is coincidental and suggests a diandric diploid nature of the molar pregnancy rather than being the cause of the molar pregnancy.

In summary, we report on a case of molar pregnancy with homozygous pericentric inversion on chromosome 9. Additional high-resolution SNP microarray confirmed whole genome isodisomy confirming the diagnosis of molar pregnancy caused by monospermy and the subsequent endoreduplication of haploid sperm resulting in diandric diploidy.

## Electronic supplementary material

Below is the link to the electronic supplementary material.

Supplementary Material 1

Supplementary Material 2

Supplementary Material 3

Supplementary Material 4

## Data Availability

No datasets were generated or analysed during the current study.

## Abbreviations

CHM: Complete hydatidiform mole
D & C: Dilation and curettage
H & E: Hematoxylin and Eosin stain
PHM: Partial hydatidiform mole
SNP: Single nucleotide polymorphism
UPD: Uniparental disomy
