# Supplementary figures and images for: Whole genome uniparental isodisomy detected using single nucleotide polymorphism (SNP) microarray in molar pregnancy: a case report
Source: Mol Cytogenet. 2025 Feb 25;18:4. doi: 10.1186/s13039-025-00707-6 (PMC11863443; doi:10.1186/s13039-025-00707-6)

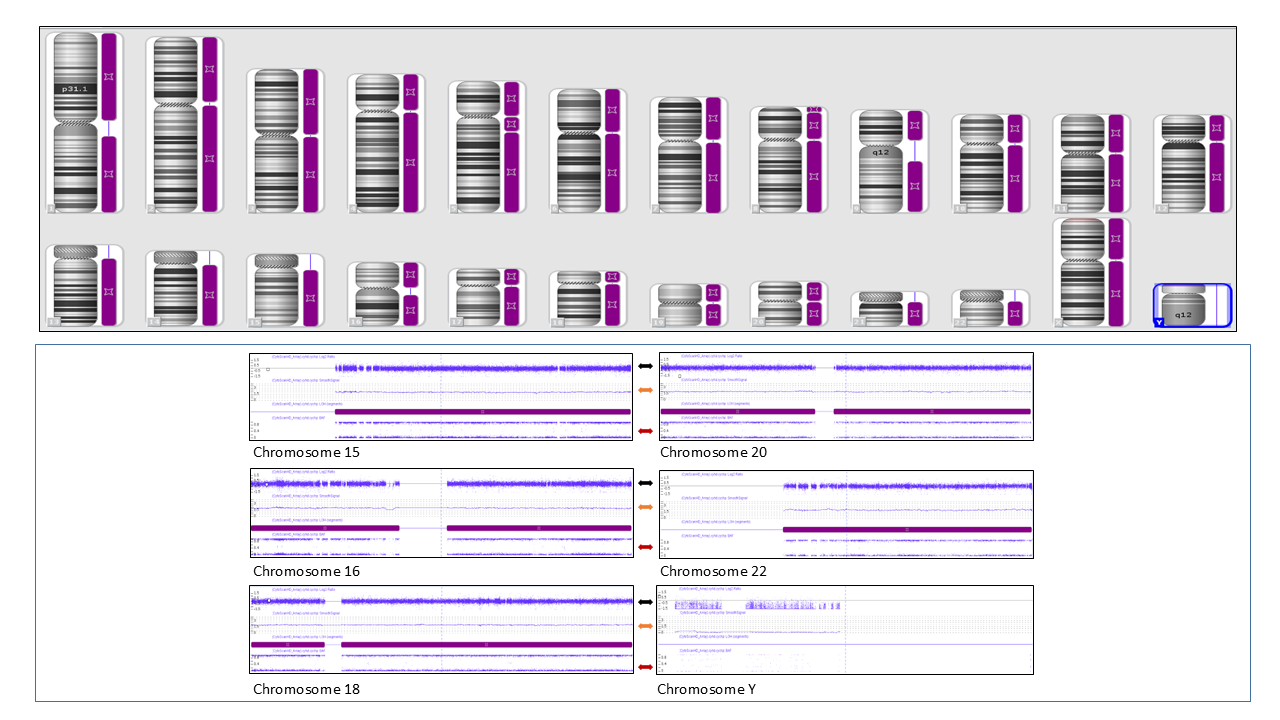

Supplement: Supplementary file 1 — Supplementary Material 1 [file 13039_2025_707_MOESM1_ESM.png]

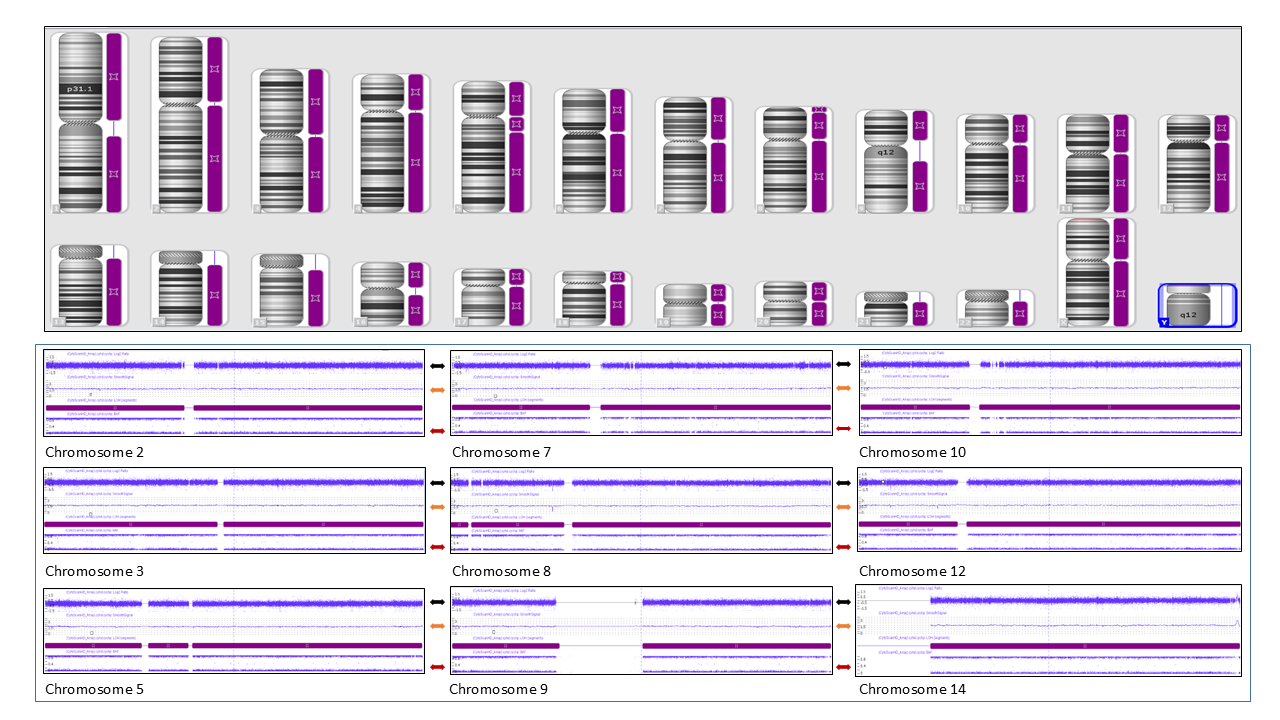

Supplement: Supplementary file 2 — Supplementary Material 2 [file 13039_2025_707_MOESM2_ESM.png]
